# Evaluating ChatGPT’s Performance in Classifying Pertrochanteric Fractures Based on Arbeitsgemeinschaft für Osteosynthesefragen/Orthopedic Trauma Association (AO/OTA) Standards

**DOI:** 10.7759/cureus.78068

**Published:** 2025-01-27

**Authors:** Mitsuaki Noda, Shunsuke Takahara, Shinya Hayashi, Atsuyuki Inui, Keisuke Oe, Takehiko Matsushita

**Affiliations:** 1 Orthopedics, Himeji Central Hospital, Himeji, JPN; 2 Orthopedics, Hyogo Prefectural Kakogawa Medical Center, Kakogawa, JPN; 3 Orthopedics, Kobe University Graduate School of Medicine, Kobe, JPN

**Keywords:** ao/ota, chatgpt, ct, fracture classification, pertrochanteric fracture

## Abstract

Introduction

Generative Pre-Training Transformer (ChatGPT) has become widely recognized for its capability to generate text, synthesize complex information, and perform a variety of tasks without requiring human specialists for data collection. The latest iteration, ChatGPT-4, is a large multimodal model capable of integrating both text and image inputs, rendering it particularly promising for medical applications. However, its efficacy in analyzing radiographic images remains largely unexplored.

Aim

This study aims to (i) address the lack of data on the accuracy of ChatGPT in radiographic fracture classification into stable or unstable under the revised Arbeitsgemeinschaft für Osteosynthesefragen/Orthopedic Trauma Association (AO/OTA) classification system, and this procedure is also performed by surgeons, and (ii) compare the agreement between surgeons or ChatGPT-based performance. The study hypothesizes that the use of ChatGPT would achieve moderate agreement with orthopedic surgeons.

Materials and methods

Patients diagnosed with pertrochanteric fractures were retrospectively collected. Patients with both preoperative two-directional plain radiographs and CT scans (3D-CT) images were conditioned for enrollment into the study.

Two orthopedic surgeons (observer 1 and observer 2, respectively) and one resident (observer 3) were once assigned to dichotomized groups into A1 (stable) or A2 (unstable) based on AO/OTA classification using two-directional plain radiographs.

Prior to the ChatGPT study, all the anteroposterior images trimmed at the fractured side, attached with figure names including gender, and age, were inputted into OpenAI ChatGPT-4.

Radiological evaluation prompts were designed to initiate ChatGPT’s classification analysis of the uploaded radiographic images. A single observer (MN) decided the classification patterns by examining 3D CT scan images as well as plain radiographs. This judgment of A1 (stable) and A2 (unstable) was set as a benchmark to mark the results of observers and ChatGPT based on plain radiographs.

Results

The cohort consisted of 29 males and 90 females, with a mean age of 87 years after the data exclusion.

The fractures were classified into A1 (stable) and A2 (unstable) groups based on CT imaging. The A1 group included 50 patients (13 males, 37 females; mean age: 86.2 ± 7.8 years), while the A2 group included 69 patients (16 males, 53 females; mean age: 87.0 ± 7.9 years).

Kappa values for fracture classification between plain radiographs evaluated by the three observers and ChatGPT, compared to the CT-based gold standard, showed fair to moderate agreement: Observer 1: 0.494 (95% CI: 0.337-0.650), Observer 2: 0.390 (95% CI: 0.227-0.553), Observer 3: 0.360 (95% CI: 0.198-0.521), and ChatGPT: 0.420 (95% CI: 0.255-0.585). ChatGPT demonstrated accuracy, sensitivity, specificity, and positive and negative predictable values comparable to the human observers, suggesting moderate reliability.

Conclusion

This study demonstrates that ChatGPT can classify pertrochanteric fractures into A1 (stable) and A2 (unstable) under the Revised AO/OTA Classification System. Its moderate agreement with CT-based assessments (κ = 0.420) is comparable to the performance of orthopedic surgeons. Moreover, ChatGPT is straightforward to integrate into clinical workflows, requiring minimal data collection for training.

## Introduction

The rising incidence of femoral pertrochanteric fractures in the elderly population is a problem in health care globally, especially in developed countries [1]. The existing guideline recommends dynamic hip screwing (DHS) for stable pertrochanteric fractures and using intramedullary nails (IMN) or a modified DHS for unstable fractures [2,3]. The meta-analysis of unstable fractures suggested this theory that the use of IMN rather than dynamic DHS would enhance functional outcomes [4,5], although their results of the fracture classification are usually based on plain radiographs with lower inter-observer reliability [6,7]. Therefore, better image-analyzing techniques like artificial intelligence (AI) for plain radiographs would be expected to improve classifying scores. The more diverse and comprehensive the data, the better the AI system can also generalize and perform in real-world scenarios, which leads to overcoming bias and improving generalizability [8,9]. However, massive dataset preparation required to train for the recognition of patterns and accurate predictions remains a burden to surgeons in practicing conventional AI models. Considering these points, it is essential to complete these groupings accurately with less data stock if surgeons hope AI will wisely support graphic classification.

Generative Pre-Training Transformer (ChatGPT), developed by OpenAI, is an AI tool that gained notable popularity for its ability to generate texts, synthesize complex information, answer questions, and interpret phrases within seconds [10]. Among several outstanding features, this AI system does not require surgeons for data gathering [11] and is trained to predict the probability of a given word sequence based on its context from an immense database through the internet, spread across several layers of complexity [10]. Additionally, the most recent version of ChatGPT-4, a large multimodal model capable of integrating text and image inputs such as plain radiographs, CT scans, MRI studies, and echographs, is of significant interest to our field [12]. Since orthopedists highly depend on these figures, AI improvement would be expected to contribute to our practice. So far, the efficacy of ChatGPT regarding its image analysis capabilities has yet to be fully determined, especially for the classification of fracture types, to our limited knowledge [13], although this method demonstrates easy access to clinical application.

This study aims to (i) address the lack of data on the accuracy of AI tools like ChatGPT in radiographic fracture classification into stable or unstable under the revised Arbeitsgemeinschaft für Osteosynthesefragen/Orthopedic Trauma Association (AO/OTA) classification system [14], and this procedure is also performed by surgeons, (ii) compare the agreement between surgeons or ChatGPT-based performance of image classifications. The current study hypothesizes that the use of the ChatGPT method would achieve moderate agreement with orthopedic surgeons and that a ChatGPT-based classification would correlate favorably with CT scan evaluation.

## Materials and methods

Data source and collection

Patients diagnosed with pertrochanteric fractures from January 2016 to December 2020 were retrospectively collected from the surgical database of Kobe University-affiliated hospitals. Patients with both preoperative two-directional plain radiographs (anteroposterior and lateral views) and CT scans (3D-CT) were conditioned for enrollment into the study. Exclusion criteria were as follows: (1) AO/OTA A3-type fractures, including those registered as subtrochanteric fractures, and (2) pathologic fractures and/or the presence of moderate or severe osteoarthritic changes.

The study was approved to use ChatGPT for data analysis exclusively for scientific purposes by the Ethics Committee of Himeji Central Hospital, to use anonymous information, and the outline of the project was posted on the homepage of our hospital (approval number 2024-050). This decision was adhered to “Guidelines for Utilization of Medical Digital Data for AI Research and Development, etc.” publicly issued by a branch section of the Ministry of Health, Labour and Welfare in September 2024.

Fracture classification by observers

**Figure 1:**
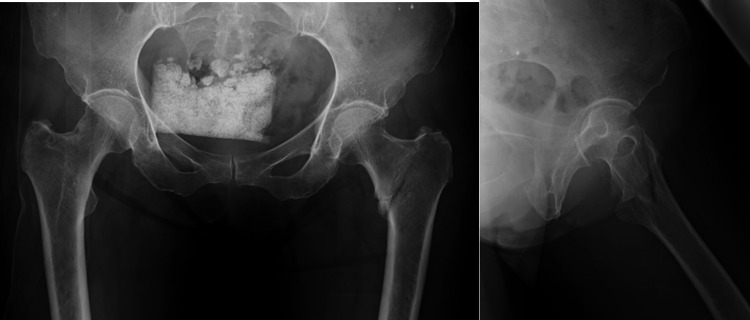
Fracture classification by observers (observers group) Three observers (two orthopedic surgeons and one resident) independently classified fractures as A1 (stable) or A2 (unstable) using two-directional plain radiographs (observers group). Observers were allowed to adjust graphic parameters (e.g., brightness and contrast) for accurate classification.

Two orthopedic surgeons observer 1 (40 years of experience) and observer 2 (over 20 years of experience), with assistance from observer 3 of the chief resident were once assigned to dichotomized groups into A1 (stable) or A2 (unstable) based on AO/OTA classification using two-directional plain radiographs. Each surgeon interpreted independently without any time restraint, magnifying and adjusting the brightness and contrast of the images for accurate identification of osseous structures. Sub-classification was unnecessary in the current study since only A1 and A2 classifications are useful in the actual clinical setting (Figure 1).

Classification through ChatGPT

**Figure 2:**
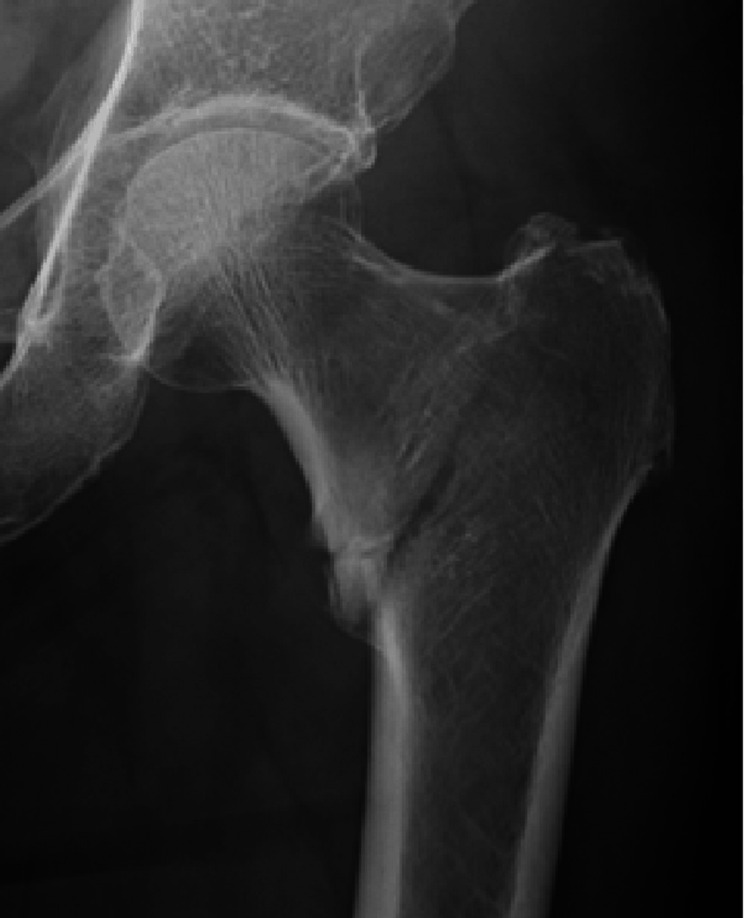
Classification through ChatGPT (ChatGPT group) ChatGPT classified fractures based on a single anteroposterior radiograph. Images were preprocessed to include only those from the proximal femur to a few centimeters below the lesser trochanter and de-identified except for age and gender.

Prior to the ChatGPT study, all the anteroposterior images trimmed into only the proximal femur at the fractured side, attached with figure names including the patients’ modified ID, gender, and age (not regarded as confidential factors under our country’s Guidelines of AI), were inputted into OpenAI ChatGPT-4 (as this is a version of the system that can analyze images). Any data that could potentially identify the patients’ individual information was removed (Figure 2).

**Figure 3:**
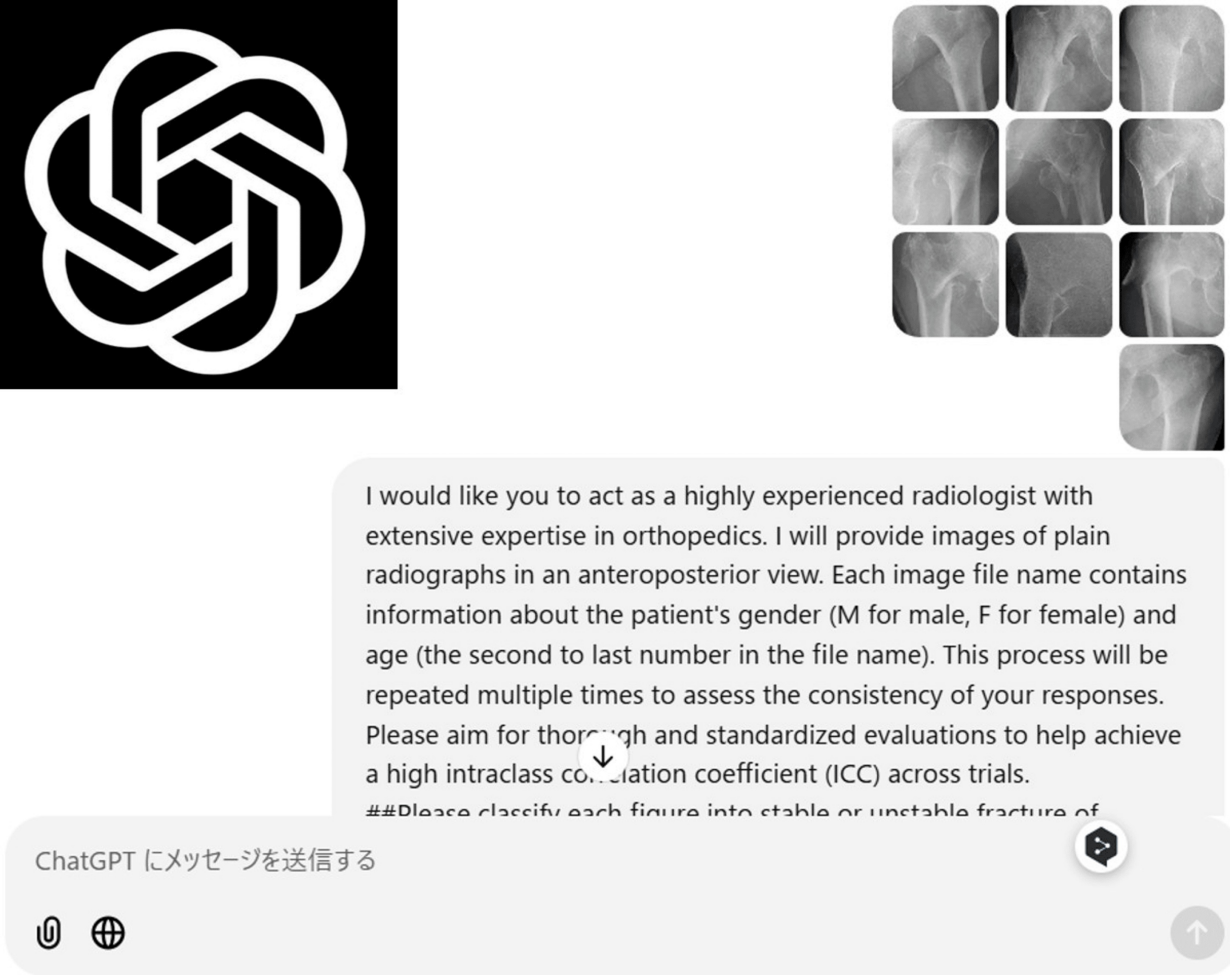
Schema of ChatGPT operation Prompt is posted to practice operation attached with 10 images. It will provide a response for each 10 images soon.

For the radiological evaluation with ChatGPT-4, the radiographic images were uploaded in bundles not exceeding 10 images, and the following standardized sequence was prompted. If ChatGPT-4 did not answer the questions adequately, the question was paraphrased or rephrased (Figure 3).

These sentences were prompted to ChatGPT to initiate analysis:

"I would like you to act as a highly experienced radiologist with extensive expertise in orthopedics. I will provide images of plain radiographs in an anteroposterior view. Each image file name contains information about the patient's gender and age in the file name. This process will be repeated multiple times to assess the consistency of your responses. Please aim for thorough and standardized evaluations to help achieve a high intraclass correlation coefficient (ICC) across trials. ##Please classify each figure into stable or unstable fracture of uploaded figures based on AO/OTA fracture classification of pertrochanteric fractures. When deciding fracture stability, sex and age are also considered besides radiographic images. You can reply to me only A1 (stable) or A2 (unstable) without any reasons. ##"

A board-certified observer 1 (MN) decided the classification patterns by examining 3D CT scan images as well as plain radiographs. This judgment of A1 (stable) and A2 (unstable) was set as a benchmark to mark the results of observers and ChatGPT based on plain radiographs.

Statistical analysis

For the statistical analysis, the study employed EZR software (Saitama Medical Center, Jichi Medical University, Saitama, Japan), a graphical interface for R software (The R Foundation for Statistical Computing, Vienna, Austria). A p-value less than 0.05 was considered statistically significant.

Univariate analyses were conducted on demographic variables, including gender, age, and fracture laterality, as classified using 3D CT scan images. The age variable was presented as means and standard deviations, treated as continuous data, and compared between the stable (A1) and unstable (A2) groups using Welch’s t-test or the Mann-Whitney U test, depending on normality. Gender and fracture laterality (left or right) were assessed using the chi-square test.

The agreement of fracture classification between the three observers and ChatGPT was examined using plain radiographs compared with CT-dependent decisions as a gold standard. Accuracy, sensitivity, specificity, positive predictive value (PPV), and negative predictive value (NPV) were calculated. Kappa coefficient values were assessed along with their corresponding 95% confidence intervals for all three observers and ChatGPT. Kappa values, showing the degree of agreement between multiple people making qualitative judgments, were interpreted as follows: poor agreement (<0.00), slight agreement (0.00-0.20), fair agreement (0.21-0.40), moderate agreement (0.41-0.60), substantial agreement (0.61-0.80), almost perfect agreement (0.81-1.00) [15].

Additionally, the confidence intervals (CI) for the kappa values, based on a sample size of 60 patients, were calculated with a lower limit of 0.5 and an upper limit of 0.8 (α = 0.05) [16].

## Results

Demographic data

**Figure 4:**
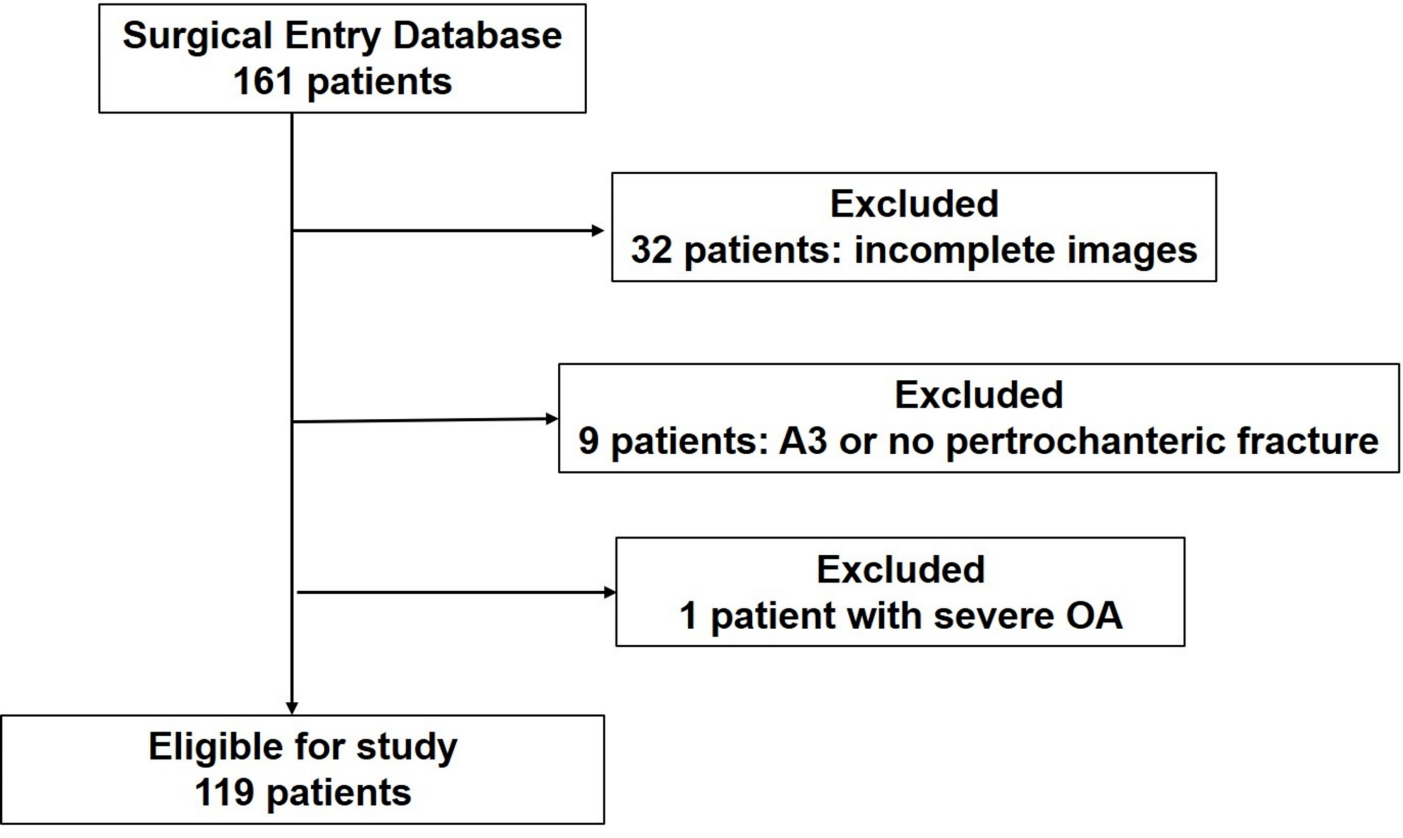
Flow diagram for patient selection A visual representation of the patient selection process. Out of 161 identified patients, 42 were excluded based on incomplete imaging, fractures classified as AO/OTA A3 or subtrochanteric, and severe osteoarthritis. The final cohort included 119 patients eligible for analysis.

From the initial 161 patients, 42 were excluded due to incomplete imaging (32 patients), fractures classified as AO/OTA A3 or subtrochanteric (9 patients), or severe osteoarthritis (1 patient). Thus, 119 patients were included in the study (Figure 4).

The cohort consisted of 29 males and 90 females, with a mean age of 87 years (range: 55-99 years). Fracture laterality was distributed as 67 left-sided and 52 right-sided.

Comparison of demographic variables

The fractures were classified into A1 (stable) and A2 (unstable) groups based on CT imaging. The A1 group included 50 patients (13 males, 37 females; mean age: 86.2 ± 7.8 years, range: 63-98 years), while the A2 group included 69 patients (16 males, 53 females; mean age: 87.0 ± 7.9 years, range: 50-99 years). There was no statistically significant difference between groups for gender, age, and laterality of fracture (Table 1). The age variable was analyzed using the Mann-Whitney U test, and gender and fracture laterality (left or right) were assessed using the chi-square test.

**Table 1:** Comparison of demographic characteristics, including gender, age, and fracture laterality, between stable (A1) and unstable (A2) groups classified by CT imaging *: p-value for differences in age between A1 and A2 groups using Welch’s t-test; **: p-values for differences in gender and laterality of fracture using the chi-square test; ^†^: mean age with standard deviation NS: not significant

Variables	A1 (stable) N = 50	A2 (unstable) N = 69	p-value
Sex:	-	-	0.72**
Male, n (%)	13 (26%)	16 (23.2%)	-
Female, n (%)	37 (74%)	53 (76.8%)	-
Age at surgery^†^ (years)	86.2±7.8 (63~98)	87.0±7.9 (50~99)	0.59*
Laterality	-	-	0.67**
Left	27 (54%)	40 (58.0%)	-
Right	23 (46%)	29 (42.0%)	-

Agreement in classification

Kappa values for fracture classification between plain radiographs evaluated by the three observers and ChatGPT, compared to the CT-based gold standard, showed fair to moderate agreement:

Observer 1: κ = 0.494 (95% CI: 0.337-0.650), observer 2: κ = 0.390 (95% CI: 0.227-0.553), observer 3: κ = 0.360 (95% CI: 0.198-0.521), and ChatGPT: κ = 0.420 (95% CI: 0.255-0.585).

The case counts from each observer and ChatGPT, along with the performance metrics for unstable fracture classification, are summarized (Tables 2-3). ChatGPT demonstrated accuracy, sensitivity, specificity, PPV, and NPV comparable to the observers, indicating moderate reliability.

**Table 2:** Case counts for each classification by observers and ChatGPT The table indicates that human observers predominantly classify cases as stable, whereas CT-based assessments favor the opposite classification. ChatGPT's classifications appear to fall between these two tendencies. Observers 1 and 2 are orthopedic surgeons, while observer 3 is a resident.

Case counts	A1 (stable)	A2 (unstable)
Observer 1	58	61
Observer 2	67	52
Observer 3	78	41
ChatGPT	55	64
CT-based assessment	50	69

**Table 3:** Agreement of fracture classification (observers and ChatGPT versus CT imaging) Summary of agreement levels between fracture classifications determined using plain radiographs (evaluated by three observers and ChatGPT) compared with CT imaging as the gold standard. Observers include two orthopedic surgeons and one resident. The table presents kappa values, confidence intervals (CIs), and performance metrics, including accuracy, sensitivity, specificity, positive predictive value (PPV), and negative predictive value (NPV), highlighting the reliability of each method. Kappa values and performance metrics indicate moderate agreement between ChatGPT and CT imaging, comparable to human observers.

	Accuracy	Sensitivity	Specificity	Positive prediction value	Negative prediction value	Kappa value (95% CI)
Observer 1	0.748	0.780	0.720	0.672	0.820	0.494 (0.337~0.650)
Observer 2	0.689	0.800	0.609	0.564	0.854	0.390 (0.227~0.553)
Observer 3	0.664	0.880	0.507	0.564	0.854	0.360 (0.198~0.521)
ChatGPT	0.714	0.700	0.720	0.648	0.769	0.420 (0.255~0.585)

## Discussion

The study proposes using ChatGPT, a generative AI model, to classify pertrochanteric fractures into A1 (stable) and A2 (unstable) under the Revised AO/OTA Classification using an anteroposterior plain radiograph. In this study, the ChatGPT analysis, based on a single plain radiograph, demonstrated moderate agreement with CT images, comparable to the results obtained by orthopedic surgeons who used two plain radiographic views. Notably, surgeons can use ChatGPT without the extensive effort of collecting and annotating large datasets for AI training.

Causative factors for the limited accuracy of ChatGPT-based image analysis

Several factors could explain the moderate accuracy of ChatGPT’s graphical analysis. First, AI performance in image interpretation may still need further development. Only a year has passed since the introduction of multimodal ChatGPT-4V, which can interpret radiological images and limited clinical data to arrive at more accurate diagnoses - an improvement from the previous version (3.5) that originated from large language models [17]. In one study, ChatGPT-4.o performed significantly worse on questions requiring image interpretation compared to text-only questions (p < 0.001) [13]. These limitations may be attributed to ChatGPT’s linguistic foundation, which was not initially designed for image-based tasks [10]. Although the latest version is more creative and can handle complex instructions [18], further refinements are necessary for optimal performance in radiology. Second, the paucity of radiographic images related to the AO/OTA classification could contribute to lower accuracy [13]. The revised AO/OTA classification was published in 2018 [14], and due to ChatGPT’s training data cutoff, it may not include a sufficient volume of radiographs from the last three years. Consequently, the limited short-term availability of relevant images likely restricts its current classification accuracy. Third, the absence of adequate lateral wall evaluations in plain radiographs - a crucial factor for classification - further limits the reliability of both AI models and human surgeons [6,7]. These inherent challenges underscore the difficulty of improving both interobserver consistency and AI performance in clinical applications.

Contribution of ChatGPT to fracture classification

As far as we know, ChatGPT for direct fracture classification is not yet ready for routine clinical use and has not been widely reported in the English literature, although marginally satisfactory outcomes were noted for distal radius fracture detection using plain radiographs [19]. Another comparative study for fracture classification relied on radiographic reports rather than the radiographs themselves, introducing variability based on subjective descriptions by radiologists [20]. Given the relatively small number of studies on ChatGPT-based image analysis, more research is needed before definitive conclusions can be drawn.

Strengths

A notable advantage of using ChatGPT is its simplicity and low burden for clinicians; it does not require a large, curated dataset for AI training because it employs a self-learning model. Before ChatGPT, surgeons had to collect thousands of radiographs, trim them appropriately, adjust image quality, and exclude suboptimal data time-consuming tasks that may hinder broader AI adoption [21,22]. The generative pre-trained approach in ChatGPT circumvents much of this workload. Another strength of our investigation is that the present study provided patient demographic data (gender and age) to ChatGPT-4o for image interpretation [17]. Studies indicate that these factors can influence fracture stability [20,23,24]. Including additional demographic information could enhance the precision of AI-based classification. Third, if this study helps healthcare providers recognize the limitations of ChatGPT in image analysis, it could lead to substantial benefits in routine clinical practice.

Limitations of current ChatGPT study

Despite its sophisticated capabilities, ChatGPT-4 does have notable limitations. First, potential biases may exist because much of the model’s data comes from high-income countries and textbooks that do not represent diverse global populations; ethical restrictions also limit healthcare-related data on the internet [11,25]. Second, ChatGPT can yield different outputs even when given identical prompts. While the current study used the first answer provided, more extensive prompt engineering and iterative refining could improve reliability [25]. Third, the training data for ChatGPT-4 remains undisclosed, making it impossible to ascertain the size or source of the original dataset [19,25]. Fourth, this study focuses solely on a binary classification (stable vs. unstable). Although the AO/OTA system includes five classes, clinical settings often find stable versus unstable sufficient.

Future direction

The study anticipates that ChatGPT will foster numerous innovative contributions in orthopedics by expanding beyond existing knowledge frameworks. For instance, future fracture classifications could integrate factors such as comminution, displacement, and osteoporosis, moving away from reliance solely on fragment counts. AI could thus facilitate the development of more nuanced and comprehensive classification systems.

Many surgeons believe ChatGPT-4o’s capabilities will continue to improve. Hirosawa et al. proposed analyzing diverse medical images, from physical examination findings to various diagnostic modalities [12]. According to their study, GPT-3 achieved 46% accuracy with zero prompting and marginally improved to 50% with more specific training and extensive prompt tuning [11]. These findings align with the current observation of ChatGPT’s evolving performance.

This study strongly believes surgeons should not see AI or ChatGPT as competitors in terms of image interpretation or memorized knowledge, especially as technological singularity approaches in this field. Instead, medical professionals should leverage their problem-solving skills, clinical reasoning, and patient interaction competencies that AI models cannot replace [26].

## Conclusions

This study demonstrates that ChatGPT, an advanced AI system, can classify pertrochanteric fractures into A1 (stable) and A2 (unstable) under the Revised AO/OTA Classification System using only an anteroposterior plain radiograph. Its moderate agreement with CT-based assessments (κ = 0.420) is comparable to the performance of orthopedic surgeons who rely on two radiographic views. Moreover, ChatGPT is straightforward to integrate into clinical workflows, requiring minimal data collection for training.

## References

[1] Chatziravdeli V, Vasiliadis AV, Vazakidis P, Tsatlidou M, Katsaras GN, Beletsiotis A (2021). The financial burden of delayed hip fracture surgery: a single-center experience. Cureus.

[2] Cho YC, Lee PY, Lee CH, Chen CH, Lin YM (2018). Three-dimensional CT improves the reproducibility of stability evaluation for intertrochanteric fractures. Orthop Surg.

[3] Rehme J, Woltmann A, Brand A, von Rüden C (2021). Does auxiliary cerclage wiring provide intrinsic stability in cephalomedullary nailing of trochanteric and subtrochanteric fractures?. Int Orthop.

[4] Zhu Q, Xu X, Yang X, Chen X, Wang L, Liu C, Lin P (2017). Intramedullary nails versus sliding hip screws for AO/OTA 31-A2 trochanteric fractures in adults: a meta-analysis. Int J Surg.

[5] Gleich J, Neuerburg C, Linhart C (2021). Inferior outcome after unstable trochanteric fracture patterns compared to stable fractures in the elderly. J Clin Med.

[6] Chan G, Hughes K, Barakat A, Edres K, da Assuncao R, Page P, Dawe E (2021). Inter- and intra-observer reliability of the new AO/OTA classification of proximal femur fractures. Injury.

[7] Zarie M, Mohamoud MF, Farhoud AR, Bagheri N, Khan FM, Heshmatifar M, Klantar H (2020). Evaluation of the inter and intra-observer reliability of the AO classification of intertrochanteric fractures and the device choice (DHS, PFNA, and DCS) of fixations. Ethiop J Health Sci.

[8] Ng MY, Youssef A, Miner AS (2023). Perceptions of data set experts on important characteristics of health data sets ready for machine learning. A qualitative study. JAMA Netw Open.

[9] Akinrinmade AO, Adebile TM, Ezuma-Ebong C (2023). Artificial intelligence in healthcare: perception and reality. Cureus.

[10] Rodrigues Alessi M, Gomes HA, Lopes de Castro M, Terumy Okamoto C (2024). Performance of ChatGPT in solving questions from the progress test (Brazilian national medical exam): a potential artificial intelligence tool in medical practice. Cureus.

[11] Kung TH, Cheatham M, Medenilla A (2023). Performance of ChatGPT on USMLE: potential for AI-assisted medical education using large language models. PLOS Digit Health.

[12] Hirosawa T, Harada Y, Tokumasu K, Ito T, Suzuki T, Shimizu T (2024). Evaluating ChatGPT-4’s diagnostic accuracy: impact of visual data integration. JMIR Med Inform.

[13] Posner KM, Bakus C, Basralian G, Chester G, Zeiman M, O'Malley GR, Klein GR (2024). Evaluating ChatGPT’s capabilities on orthopedic training examinations: an analysis of new image processing features. Cureus.

[14] Meinberg EG, Agel J, Roberts CS, Karam MD, Kellam JF (2018). Fracture and dislocation classification compendium-2018. J Orthop Trauma.

[15] Koo TK, Li MY (2016). A guideline of selecting and reporting intraclass correlation coefficients for reliability research. J Chiropr Med.

[16] Klaber I, Besa P, Sandoval F, Lobos D, Zamora T, Schweitzer D, Urrutia J (2021). The new AO classification system for intertrochanteric fractures allows better agreement than the original AO classification. An inter- and intra-observer agreement evaluation. Injury.

[17] Zhu L, Mou W, Lai Y (2024). Step into the era of large multimodal models: a pilot study on ChatGPT-4V(ision)'s ability to interpret radiological images. Int J Surg.

[18] (2024). OpenAI. https://openai.com.

[19] Mert S, Stoerzer P, Brauer J (2024). Diagnostic power of ChatGPT 4 in distal radius fracture detection through wrist radiographs. Arch Orthop Trauma Surg.

[20] Russe MF, Fink A, Ngo H, Tran H, Bamberg F, Reisert M, Rau A (2023). Performance of ChatGPT, human radiologists, and context‑aware ChatGPT in identifying AO codes from radiology reports. Sci Rep.

[21] Sato Y, Takegami Y, Asamoto T (2021). Artificial intelligence improves the accuracy of residents in the diagnosis of hip fractures: a multicenter study. BMC Musculoskelet Disord.

[22] Urakawa T, Tanaka Y, Goto S, Matsuzawa H, Watanabe K, Endo N (2019). Detecting intertrochanteric hip fractures with orthopedist-level accuracy using a deep convolutional neural network. Skeletal Radiol.

[23] Mattisson L, Bojan A, Enocson A (2018). Epidemiology, treatment and mortality of trochanteric and subtrochanteric hip fractures: data from the Swedish fracture register. BMC Musculoskelet Disord.

[24] Noda M, Takahara S, Inui A, Osawa S, Matsushita T (2023). Inter-observer agreement and reproducibility of pertrochanteric fracture classification using plain radiograph versus computed tomogram images: a study of 523 patients. Cureus.

[25] Gao CA, Howard FM, Markov NS, Dyer EC, Ramesh S, Luo Y, Pearson AT (2023). Comparing scientific abstracts generated by ChatGPT to real abstracts with detectors and blinded human reviewers. NPJ Digit Med.

[26] Mbakwe AB, Lourentzou I, Celi LA, Mechanic OJ, Dagan A (2023). ChatGPT passing USMLE shines a spotlight on the flaws of medical education. PLOS Digit Health.

